# DNA double-strand–break complexity levels and their possible contributions to the probability for error-prone processing and repair pathway choice

**DOI:** 10.1093/nar/gkt556

**Published:** 2013-06-25

**Authors:** Agnes Schipler, George Iliakis

**Affiliations:** Institute of Medical Radiation Biology, University of Duisburg-Essen Medical School, 45122 Essen, Germany

## Abstract

Although the DNA double-strand break (DSB) is defined as a rupture in the double-stranded DNA molecule that can occur without chemical modification in any of the constituent building blocks, it is recognized that this form is restricted to enzyme-induced DSBs. DSBs generated by physical or chemical agents can include at the break site a spectrum of base alterations (lesions). The nature and number of such chemical alterations define the complexity of the DSB and are considered putative determinants for repair pathway choice and the probability that errors will occur during this processing. As the pathways engaged in DSB processing show distinct and frequently inherent propensities for errors, pathway choice also defines the error-levels cells opt to accept. Here, we present a classification of DSBs on the basis of increasing complexity and discuss how complexity may affect processing, as well as how it may cause lethal or carcinogenic processing errors. By critically analyzing the characteristics of DSB repair pathways, we suggest that all repair pathways can in principle remove lesions clustering at the DSB but are likely to fail when they encounter clusters of DSBs that cause a local form of chromothripsis. In the same framework, we also analyze the rational of DSB repair pathway choice.

## INTRODUCTION

The defining feature of a double-strand break (DSB) as DNA lesion is the associated disruption of molecular continuity. The DSB severs in two fragments a linear DNA molecule and linearizes a circular molecule by disrupting the sugar–phosphate backbone on both strands and at sites located directly opposite each other—or just a few nucleotides apart (up to ∼10 bp).

DSBs, by affecting both DNA strands, compromise the fundamental principle used for the repair of lesions confined to one DNA strand: the possibility to use the complementary, undamaged strand as template to restore sequence in the damaged strand. Indeed, excision-based repair pathways, such as base excision repair (BER), nucleotide excision repair and mismatch repair, use the undamaged strand as template to restore the DNA molecule after removal (excision) of the damaged, or mismatched, segment (1).

**Figure 1.**
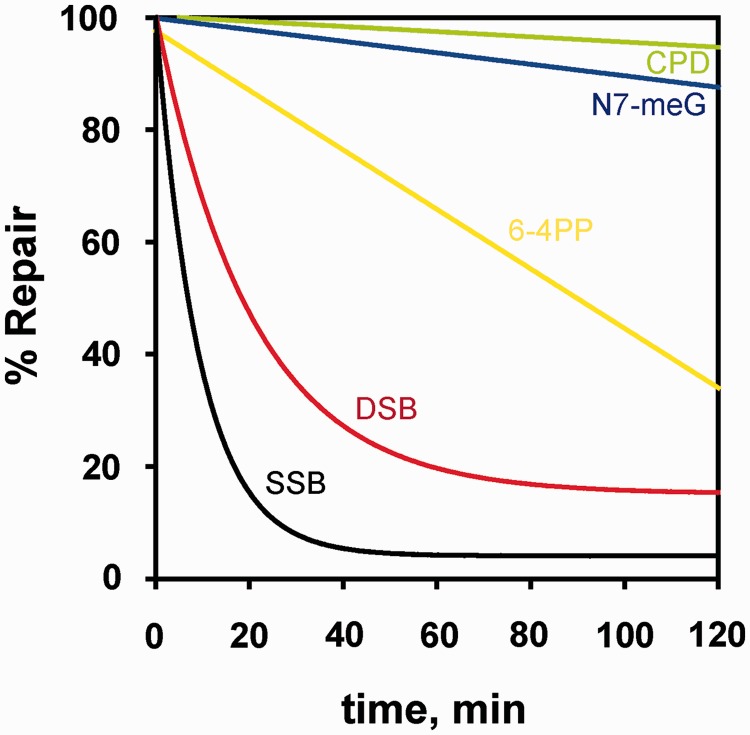
Kinetics of repair of different types of DNA lesions. Shown is the kinetics of removal from CHO-AA8 cells of SSBs, DSBs, 6–4 photoproducts (6–4PP), cyclobutane pyrimidine dimers (CPD) and, for human lymphocytes, of N7-meG. SSB and DSB repair was measured after exposure to 7.5 Gy and 100 Gy of γ-rays, respectively. SSBs were assayed by alkaline filter elution at pH 12.1 and DSBs by non-denaturing filter elution at pH 9.6 (2). Repair of UV-induced CPD and 6–4PP was measured in CHO cells by radioimmunoassay using damage-specific antibodies. Removal of antibody-binding sites after various repair times was determined after 10 J/m^2^ UV-irradiation (3). Repair of N7-meG was measured in human lymphocytes after treatment with alkylating agents (7).

This feature of the DSB allows the inference that its repair will be difficult, inherently inefficient and slow. However, comparison of the DSB repair kinetics with the kinetics measured for the repair of forms of DNA lesions only affecting one DNA strand provides a surprising outcome. Thus, CHO cells repair DSBs markedly faster than base damage or ultraviolet (UV)-induced lesions (Figure 1). Only the biologically much less consequential single-strand break (SSB) is repaired with slightly faster kinetics. Similar results can be compiled for other experimental systems and demonstrate that cells of higher eukaryotes have evolved an impressive capacity for removing DSBs from their genomes, despite the expected difficulties in performing this task.

The apparently effortless removal notwithstanding, DSBs remain biologically highly dangerous DNA lesions. Indeed, among DNA lesions, DSBs have the highest per lesion probability of causing numerous adverse biological effects including cell death, mutation, as well as transformation to a carcinogenic state.

The severity of the DSB as DNA lesion is evolutionarily ingrained into cellular function. This is convincingly demonstrated by the evolutionarily conserved, highly elaborate and complex network of responses cells mount, when detecting a DSB. The so called ‘DNA damage response (DDR)’ (8), originates, directly or indirectly, from the DSB (and single-stranded DNA regions) and includes comprehensive intracellular and intercellular regulatory processes that modify nearly every metabolic activity of the cell. The responses integrated in the DDR alert the cell to the DSB presence and set the stage for processing, adaptation or programmed cell death. Indeed, defects in DDR are associated with various developmental, immunological and neurological disorders and are a major driver of cancer (9).

The DDR is triggered not only by accidental DSBs randomly generated in the genome by exogenous agents such as ionizing radiation (IR) and certain chemicals, or during DNA replication stress (4–6), but also by programmed DSBs arising in well defined locations in the genome during meiosis, as well as during V(D)J and immunoglobulin heavy chain class switch recombination (CSR) (10). Thus, DDR integrates the biological responses initiated by DSBs into the cellular life cycle.

## DSB PROCESSING CARRIES HIGH RISK FOR MISREPAIR

It may seem surprising why a lesion that can be processed by the cell efficiently and for which the cell devotes extensive resources still remains highly dangerous and linked to severe adverse biological consequences. Extensive work carried out over the past several decades converges to the idea that the adverse consequences of DSBs mainly result from errors or accidents in their processing. Indeed, there is evidence that the probability of processing errors is for DSBs much higher than for lesions confined to one DNA strand (11–14).

Considering the nature of the DSB, three scenarios for errors can be envisioned. First, processing is somehow interrupted, the DSB remains open and the ends drift apart becoming inaccessible to each other for rejoining. Second, processing of the DSB occurs but after repair the junction is altered—slightly or severely. Associated consequences include here deletions involving several nucleotides; however, numerically conservative alterations in nucleotide sequence, as well as *de novo* additions of nucleotides are also possible (15,16). It should be noted, though, that point mutations are rare after exposure to DSB inducing agents. We discuss later that depending on the pathway engaged to the repair of a DSB, this type of error can be highly unlikely or common.

Third, processing of the DSB occurs, but during repair, incongruent ends are joined together causing thus structural alterations in the genome that can be visualized either as chromosome aberrations (mainly inter and intra chromosomal exchanges) (11–14,17–20), as size alterations in defined genomic restriction fragments after separation by gel electrophoresis (21,22), or finally as genomic alterations detected by next-generation sequencing approaches (23). This is by far the most consequential level of DSB-processing failure, as it generates new sequence combinations in the genome that disrupt or deregulate genes, and which may generate structural chromosome alterations that are incompatible with normal mitotic division. Under certain conditions, this form of error may also follow the events described in the first scenario.

**Figure 2.**
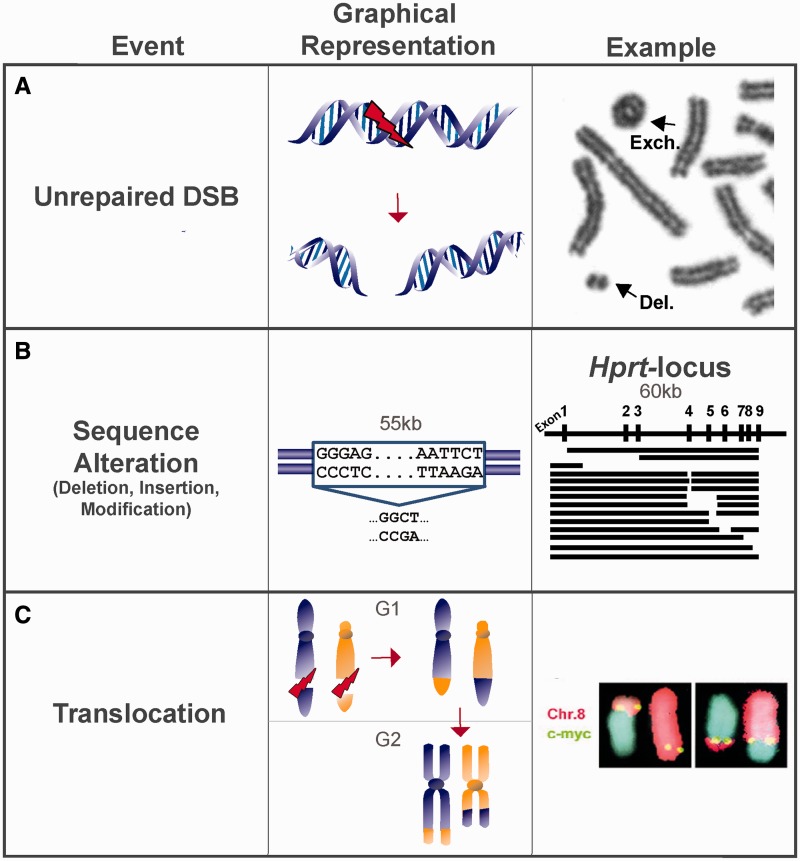
Three scenarios of DSB misrepair. (**A**) DSB ends drift apart resulting in a chromosomal aberration in the form of an acentric fragment (Del). (**B**) Rejoining of the DSB occurs but the junction is altered. Examples for large deletions in the *Hprt* locus are shown. The nine exons of *Hprt* are indicated at the top of the right panel. Genomic regions amplified by polymerase chain reaction are shown by solid lines. Spaces between the lines represent DNA sections that are deleted [drawn from results published by (24)]. (**C**) Joining of incongruent ends can cause chromosomal translocations. Fluorescence *in situ* hybridization analysis shows a c-myc/Ig locus translocation between chromosomes 8 and 14 in a multiple myeloma cell line [image from (25)]. An exchange-type aberration in the form of a ring chromosome is also shown in panel A (Exch).

Experimental evidence for all three error scenarios is abundant and typical examples are shown in Figure 2. Thus, unrepaired DSBs can surface as chromatid or chromosome breaks in the subsequent metaphase (Figure 2A); error-prone repair events can lead to large losses of sequence information inactivating a gene, for example, the HPRT gene (Figure 2B). Finally, the joining of wrong ends can cause translocations that can kill cells or can transform them to cancer cells (Figure 2C and the ring chromosome in 2A).

## SOURCES OF DSB PROCESSING ERRORS AND PROCESSING ACCIDENTS

The causes of the aforementioned described types of erroneous DSB processing events warrant discussion. Of particular importance and relevance is, without any doubt, the identification and characterization of parameters determining the probability of their occurrence. Available information on the mechanisms underpinning DSB processing allows the definition of three main sources of DSB processing errors:

1. Inherent limitations of repair pathways engaged in DSB processing. As we briefly describe later in the text, multiple pathways process DSBs, and each shows distinct and frequently inherent propensities for errors. Notably, and possibly unexpectedly, the propensity for errors can vary dramatically among repair pathways. It follows that depending on the repair pathway choice made for a particular DSB, the associated risk for errors will vary accordingly. These limitations are compounded by the specifics of the 3D organization of the genome that define the form of possible errors (e.g. translocations) on the basis of proximity between interacting regions and possibly other parameters as well (26–28).

2. The nature of the initiating DSB. Although the DSB is defined as a rupture in the double-stranded DNA molecule that can occur without chemical modification in any of the constituent DNA building blocks, it is recognized that this form of DSB is relatively rare and restricted to certain biologically induced DSBs (see later in the text). DSBs generated by physical or chemical agents can include with the DNA rupture a spectrum of chemical alterations (DNA lesions) in the neighboring bases. It is now widely considered that chemical alterations accompanying the DSB may be determinants of the form of DSB processing chosen by the cell and the probability that errors will occur during this processing. The term complexity is frequently used to describe some of these characteristics of the DSB (29,30).

3. The localization of the DSB in chromatin. The term ‘location’ can refer to the condensation status of chromatin at the site of the DSB, with two extremes: localization in euchromatin or in heterochromatin (31,32). However, location can also refer to sites with DSBs, where DNA replication or transcription occurs; these processes can be affected by a DSB, but they can also interfere with DSB processing and thus cause errors (33). Finally, location can also refer to specific characteristics of the genome including coding, or repetitive regions, intron/exon distribution, induction in active versus inactive genes and so forth.

In the following sections, we discuss possible sources of DSB processing errors and accidents. We first focus on structural aspects of the DSB, define levels of DSB complexity and discuss how DSB complexity may interfere with processing to cause errors. Subsequently, we describe briefly repair pathways engaged in DSB processing and discuss how processing errors such as those summarized in Figure 2 can emerge from their limitations. The possible role of DSB localization, within chromatin etc., in the erroneous processing of a DSB is not subject of the review.

## SYSTEMATIC ANALYSIS OF DSB COMPLEXITY LEVELS

Here, we attempt a classification of DSBs on the basis of increasing complexity and analyze how the specific characteristics of each class affect the processing requirements and the probability of processing errors. To facilitate presentation and subsequent discussion, we will define categories (types) comprising DSBs with progressively increasing complexity.

### Type 1 (T1) DSBs: the simplest form

**Figure 3.**
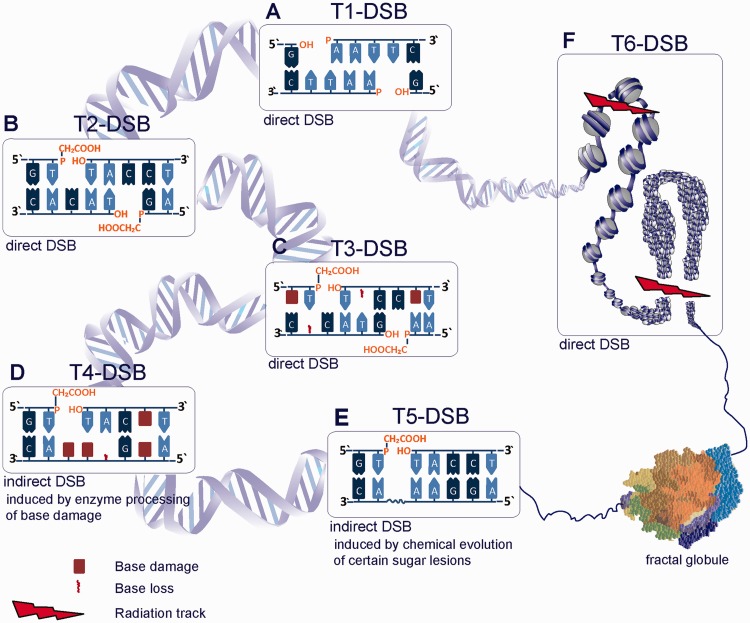
Illustration of the different types of DSBs as defined in the text. (**A**) T1-DSBs are direct DSBs induced by RE. An example for EcoRI DSB is shown that produces staggered ends with a 5′-phosphate and a 3′-OH group. (**B**) T2-DSBs are induced by IR and frequently comprise a 3′-phosphoglycolate and a 5′-OH at the DNA ends as shown in this example. (**C)** IR also induces clustered lesions from ionization clusters, defined as T3-DSBs. In this case, the direct DSB is accompanied by other types of lesions, like base damage or base loss proximal to the DSB. (**D**) T4-DSBs represent a non-DSB damage cluster that can convert to DSBs (indirect DSB) by enzymatic processing of the constituent base lesions. (**E**) T5-DSBs are also induced indirectly, up to 1 h after IR, by temperature-sensitive chemical processing of damaged sugar moieties opposing SSBs. (**F**) T6-DSBs are composed of clustered DSBs that can destabilize chromatin. Two possible scenarios are illustrated: in the first scenario (upper left) radiation induces two DSBs in the linker regions between a nucleosome risking nucleosome loss. The second scenario (lower right) shows higher-order packaging of nucleosomes forming a chromatin loop that is broken as shown by a radiation track. Here, loss of a larger segment of chromatin is possible. In the lower right corner of the drawing the 10-nm chromatin fiber is shown, compacted as a fractal globule (36,37); the opening of a loop from this fractal globule is indicated.

Classic examples of DSBs are those generated by restriction endonucleases (RE) (34,35). This family of proteins binds as a homodimer to specific DNA sequences and disrupts the phosphodiester bonds on both strands of the DNA molecule to generate either blunt or staggered ends (Figure 3A). As disruption of the phosphodiester bond by RE retains the 5′-phosphate and 3′-OH groups at each strand end, rejoining by simple ligation is in principle possible.

RE generates the simplest possible form of DSB, as they disrupt the continuity of the DNA molecule without chemically altering any of its constituent moieties, i.e. lesions in the form of sugar or base modifications are not introduced. We will term here this form of DSB type 1, T1-DSB, to distinguish it from more complex forms that are described later in the text (Figure 3A). Notably, even this ‘simple’ form of DSB is highly toxic, as indicated by the fact that RE evolved in bacteria as a defense mechanism against invading genomes.

The proposed, one-class grouping of RE-induced DSBs is certainly an oversimplification, as it disregards characteristics that may affect processing. Thus, type (3′- or 5′-) or length of protruding ends have been shown to affect the efficiency of DSB processing *in vitro*, and blunt-ended DSBs are generally more difficult to ligate than DSBs with protruding matching ends (16,38,39).

RE are frequently used as model reagents to generate DSBs at specific sites of a DNA molecule and to analyze the associated cellular responses. This approach has gained ground with the introduction of rare cutting RE and the I-SceI homing endonuclease for which the recognition sequence (18 bp) is not present in mammalian cells but can be introduced according to a pre-conceived design using molecular biology approaches (40–43). These sites can be subsequently cut to generate a DSB by either transfecting into cells vectors expressing I-SceI or by forcing the translocation from the cytoplasm into the nucleus of constitutively expressed I-SceI (44,45). The advantage of this approach is that DSBs are generated at a defined location in the genome, and appropriately constructed reporters allow functional analysis of specific repair pathways.

### Type 2 (T2), DSBs: complexity deriving from modified ends

When DSBs are induced by physical or chemical agents, the alterations generated in the DNA are more complex. Among physical agents inducing DSBs, IR takes a prominent place. This is because IR, at low doses, is present in the environment and frequently used in diagnostic medicine. At higher doses, IR is used for the treatment of human diseases like cancer and inflammation (46,47). Recently, IR has gained ground in all fields of biology as a model agent for DSB induction owing to its unique physical characteristics that allow a timely well-defined DSB induction (most DSBs are generated only during the few minutes of exposure) with even distribution within cells (48,49). This goal cannot be achieved with DSB-inducing drugs, which need time to cross cell membrane, be metabolically activated (occasionally) and reach the DNA. In addition, drugs act subsequently for extended and difficult to precisely define periods. Incidentally, similar limitations apply to RE-induced DSBs (see earlier in the text)*,* which require transfection and expression, or at a minimum intracellular translocation, of the I-SceI, or other endonuclease. Moreover, the enzyme remains functional in the cell nucleus for periods that are difficult to accurately define or precisely limit.

But why is IR generating DSBs in the DNA, and how do IR-induced DSBs compare with RE-induced DSBs? Although IR is frequently thought of as a DSB inducing agent, it by no means only generates DSBs in the DNA of irradiated cells. Actually, IR in the form of X-rays or γ-rays frequently used in the laboratory, induces, through oxidation reactions (either direct loss of an electron from DNA constituents or an attack by an ^•^OH produced by the radiolysis of adjacent water), a wide spectrum of lesions including sugar and base damages each of which outnumbers DSBs by ∼20:1 (50,51). Certain forms of sugar damages disrupt the phosphodiester backbone of the DNA molecule and produce SSBs. It is the coincidence of two SSBs in opposite DNA strands with a maximum displacement of up to 10 bp that is thought to generate DSBs. These DSBs differ from those induced by RE because they frequently comprise a 3′-damaged sugar in the form of phosphoglycolate and a 5′-OH (Figure 3B) (52,53). This form of ends precludes direct DNA ligation and necessitates end processing as a step during repair (54). We will, therefore, term this more complex form of DSB type 2, T2-DSB, to distinguish it from that induced by RE. As IR-induced DSBs are generated by coincidence of two SSBs that can also be displaced by up to 10 bp, blunt ends or ends with protruding single strands similar to those described for RE can be generated.

Oxidation reactions, similar in principle to those initiated by IR, are also initiated by H_2_O_2_, an oxidative agent that is also produced intracellularly as byproduct of the cellular metabolism (55). In this case, ^•^OH radicals, generated in the presence of metal ions by Fenton reactions, attack the DNA molecule producing base damages, SSBs and DSBs, more or less, randomly (55–57). Notably, however, after treatment with H_2_O_2_, base and sugar damages outnumber DSBs not only by 20:1 but by >10 000:1 (55). This difference in the relative yields of DSBs hints to specific characteristics of IR that underpin the efficient induction of DSBs. But what are these characteristics?

**Figure 4.**
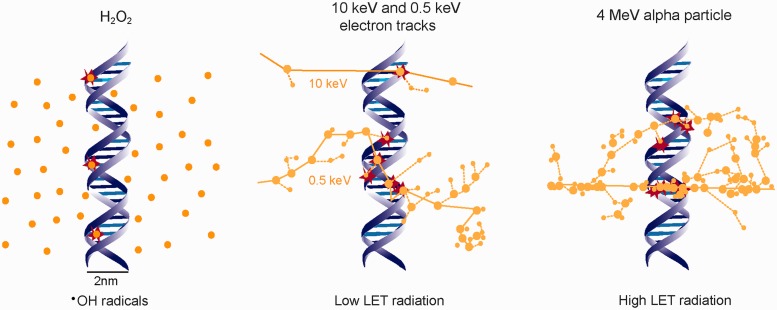
Distribution of DNA damage inducing events after exposure to H_2_O_2_ and IR of low and high LET^·^OH radicals from H_2_O_2_ are evenly distributed in space and induce, therefore, also evenly distributed DNA damage. In the case of IR, ionization events localize along the particle tracks [middle panel 0.5 and 10 keV electrons (e^−^), right panel 4 MeV α particle] and can, therefore, induce clustered damage as indicated. Note that with increasing LET (from 10 to 0.5 keV e^−^ up to the 4 MeV α particle) the damage clustering increases. Large dots represent ionizations and small dots represent excitations along the radiation track. Monte Carlo simulated tracks are drawn for the 0.5 keV e^−^ and the α particle on the same scale as the DNA [redrawn from (58)]. The track for the 10 keV e^−^, as well as the events shown after treatment with H_2_O_2_ are by free drawing and shown only for illustration purposes.

After exposure to H_2_O_2_, and depending on the concentration used, the oxidation events generated by ^•^OH radicals are relatively evenly distributed within the cell and the DNA and produce large amounts of SSBs (55–57). They produce low yields of DSBs because the probability of simultaneous local induction of two SSBs in opposite DNA strands is very low from a random distribution of oxidation events (Figure 4). However, after exposure to IR, the ionization events causing DNA damage, either directly by occurring in the DNA molecule itself or indirectly through radicals produced by ionization of atoms or molecules in the vicinity of the DNA, are not evenly distributed in space but localize along the tracks of the ionizing particles- secondary electrons in the case of X-rays and γ-rays (59). Using computational approaches based on Monte Carlo track structure codes the stochastic patterns of ionization can be computed (49,60,61). These calculations show that secondary electrons, at the end of their tracks, generate clusters of ionizations, i.e. multiple ionizations confined in a small volume. When such ionization clusters are generated within the DNA, they can induce damages on both DNA strands and thus give rise to DSBs (Figure 4, see track of the 0.5 keV electron).

It is widely accepted that the adverse biological effects of X-rays or γ-rays derive from DSBs generated within such ionization clusters (62,63), rather than by the coincidence of independently generated ionizations on opposite DNA strands. This is the reason why the dose-yield curves for DSBs increase linearly and not with the square of the applied radiation dose. The simplest DSB that can be generated within such an ionization cluster is a T2-DSB (Figure 3B).

### Type 3 (T3), DSBs: complexity deriving from the presence of DNA lesions in the vicinity of the break

Despite the generation of ionization clusters at the ends of low energy electron tracks, X-rays and γ-rays still deposit 50–70% of their energy in well-separated ionization events from high-energy electrons that ionize sparsely and generate a relatively even ionization pattern within the cell (compare the tracks of high- and low-energy electrons in Figure 4) (62,63). This is why X-rays and γ-rays are considered sparsely ionizing, or low-linear energy transfer (LET), forms of IR. On the other hand, particulate forms of ionizing radiation such as neutrons, α particles, or carbon ions, are considered densely ionizing, or high LET, forms of radiation because they ionize along their tracks at a higher rate than the electrons generated by X-rays (64).

The computed ionization patterns in Figure 4 show the increased ionization density generated by an α particle as compared with an X-ray-generated secondary electron (particularly the high energy one). This increased clustering will also generate frequently DNA damage that is more complex than that induced by low-LET radiations, in the sense that it will comprise more lesions within one or two turns of the DNA helix. It constitutes what is sometimes called clustered damage sites (CDS) or multiply damaged sites (50,65). Although CDS is generated by low-LET radiation, such as X-rays, it occurs more frequently after exposure to high-LET radiations and is implicated in their enhanced biological effects. This is particularly important if one considers that similar numbers of ionizations, and thus presumably also DNA lesions, are generated after exposure to high- and low-LET radiations (51,65–67). Evidently, not only the number of ionizations but also their spatial distribution determines the biological effects of IR (49).

Indeed, although only ∼30% of DSBs are expected to contain lesions in addition to the two strand breaks after exposure to low-energy electrons, this fraction increases to 70% after exposure to α-particles. Also, the ratio of the number of SSBs to DSBs is decreased from 22.8 for ^60^Co γ-rays to 3.4 for 50 MeV ^12^C-ions (30,47). As these changes do not increase the yields of DSBs in a manner corresponding to the increased killing after exposure to high- versus low-LET radiation, it can be inferred that increased clustering of DNA damage is an important determinant of the gravity of the resulting biological effect (but see also later in the text) (68).

The simultaneous presence of DSBs and other forms of DNA damage within a clustered damage site generates the next level of complexity, which we term here DSB of type 3, T3-DSB (Figure 3C). The increased complexity of T3-DSBs may compromise cellular repair through the simultaneous recruitment and even engagement of two repair pathways (e.g. DSB repair and BER) to lesions present in close proximity in the DNA molecule. A similar situation is generated by the covalent attachment of proteins at the DSB ends, as it occurs, for example, in DSBs induced by topoisomerase inhibitors (69). Such complications may increase the probability of processing errors as compared with the simpler forms of DSBs described later in the text.

### Type 4 (T4) DSBs: indirect form, arising from base damage processing within a non–DSB-CDS

In addition to DNA damage clusters that generate DSBs right at the outset, IR also generates clusters of base damage, possibly including SSBs, which do not form DSBs immediately (non-DSB clusters). DSBs can subsequently form through the processing of a base lesion opposite an unrepaired SSB, or through the parallel processing on both DNA strands of base damage (Figure 3D) (30,70–72). There is evidence that this form of clustered DNA damage outnumbers T2/T3-DSBs after exposure to low-LET radiation by nearly 4:1.

Although the extremely fast processing of SSBs and the particularly slow processing of base damage (Figure 1) reduce the probability for unrepaired SSBs when BER starts, here again repair by either pathway may be impaired by this clustering of DNA damage and possibly also by the parallel recruitment of components of different repair pathways. Indeed, the repair efficiency of non-DSB clusters processed by BER depends on the nature, the orientation (bi-stranded or tandem) and the distance between lesions (73–76). One or more lesions within a non-DSB cluster can remain unrepaired as a result of reduced or altered glycosylase activity in this context. In the case of bi-stranded clusters containing either two AP sites or an SSB opposing an AP site, a DSB is likely to form through the incision of the AP site during repair (77).

Indirect DSBs forming by the simultaneous disruption of the phosphodiester bond at base damage sites in opposite DNA strands or with the combination of BER activity with a SSB at the opposite strand, form yet another level of complexity that integrates the parameter time post-irradiation in the induction process, and which we will, therefore, term here type 4 DSBs, T4-DSBs. A discriminating characteristic of a T4-DSB is that as it forms, proteins participating in SSBR and/or BER may already be engaged at or near the ends of the resulting DSB, which may impair its recognition and processing by the cell.

### Type 5 (T5) DSBs: indirect form arising from chemical processing of sugar damage within a CDS

There is evidence that IR induces, in addition to sugar lesions promptly disrupting the sugar–phosphate backbone (prompt DSBs), also lesions doing so after temperature-dependent chemical processing (delayed DSBs) (78). These thermally labile sugar lesions constitute what are considered radiation-induced labile sites (68,78,79). They can include diverse forms of sugar damage, abasic sites and forms of base damage affecting sugar stability. Chemical evolution of such lesions to SSBs within a CDS can generate additional DSBs (62,78,80–83).

Until recently, it was believed that in mammalian cells evolution of such lesions to DSBs only occurs when DNA is incubated after irradiation at high, non-physiological temperatures (e.g. ∼50°C typically used for cell lysis to analyze DNA breakage) (84–86). However, recent work (87–89) provides evidence that IR induces thermally unstable lesions, which evolve within ∼1 h under physiological temperatures to SSBs and contribute, when present within a CDS, to the formation of DSBs. These delayed-forming DSBs are thought to be generated continuously during the first post-irradiation hour, and to add to promptly induced DSBs (88).

This process represents yet another way for generating indirectly DSBs within a CDS, which we will term here type 5 DSBs, T5-DSBs, to distinguish them from the other categories described above and also later in the text (Figure 3E). Like T4-DSB, T5-DSBs evolve from non-DSB CDS and belong, therefore, to the indirectly induced DSBs. Several of the complications outlined for the processing of T4-DSBs also apply for the processing of T5-DSBs.

### Type 6 (T6) DSBs: complexity deriving from destabilizing chromatin fragmentation via multiple, clustered DSBs

As an additional level of increasing DSB complexity, we consider here clusters of DSBs, where the individual DSBs can in principle belong to any of the aforementioned defined types. This form of DNA damage disrupts the continuity of the DNA in the same general way as simpler forms of DSBs do. However, by involving several DSBs in close proximity (DSB clusters), it severely undermines local chromatin stability and thus overall processing in a location- and composition-dependent manner. On the basis of its constitution, this form of damage can also be considered as a form of highly local chromothripsis—a phenomenon whereby as of yet undefined processes cause extensive local genomic fragmentation (thripsis), which invokes inaccurate rejoining that feeds carcinogenesis (90–93).

DSB clustering as a source of small DNA fragments in irradiated cells and a cause of irreversible radiation effects has been considered by several investigators [see (49) for a review]. Bryant, Johnston and colleagues (94–96) developed a non-ionic neutral filter elution assay to generate histone-depleted nuclear structures retaining higher-order nuclear matrix organization, and used it to measure DNA fragment loss from two or more DSBs within a single-looped chromatin domain. They observed that the spatial distribution of DSBs in higher-order chromatin loops affects their reparability. Fast repair is measured in loops containing a single DSB but slow repair in loops containing multiple DSBs. The latter form of repair is not detectable in cells deficient in Ku80 (see next section). They proposed that higher-order chromatin structure and the spatial distribution of DSBs in topologically independent, looped domains (of ∼1.6 Mb, as in replicon clusters) plays a crucial role in DSB repair and that misrepair involves DNA fragments loss at such DSB clusters.

Holley and Chatterjee (97) also considered DSB clusters as a particularly consequential form of radiation damage and performed Monte Carlo simulations for the induction in chromatin of such clusters with increasing LET. Their calculations confirm the overall increase in DSB clustering with LET and show the potential of generating in this way relatively small DNA fragments. In these calculations, fragmentation peaks are found at 85 bp and then again at multiples of 1000 bp, independently of LET, possibly representing the revolution period of the DNA about the histone core (∼85 bp) and the periodicity of nucleosomes packed in a solenoid model of chromatin (see later in the text), respectively. Notably, such small fragments can indeed be detected experimentally using pulsed-field gel electrophoresis in irradiated human fibroblasts (98,99) and can also be inferred by alternative modeling approaches (49,100,101). Atomic force microscopy imaging also shows the induction of clustered DSBs and the associated formation of short DNA fragments- even when irradiating ‘naked’ DNA devoid of any organization as chromatin (102). In the latter experiments, only 35% of the generated fragments are smaller than 147 bp in length after exposure to low LET radiation, but this proportion increases to 70% after exposure to high-LET radiation.

Small (<70 bp) DNA fragments generated from clustered DSBs have also been implicated by Wang *et al.* (103) in the enhanced killing observed after exposure of cells to high-LET radiation. The authors attribute the enhanced toxicity of such fragments specifically to their inability to accommodate bi-directional binding of the Ku-protein (requires ∼30 bp on each side of the DNA fragment, see later in the text and Figure 6D), which is required for the efficient repair of the DSBs within the cluster (103). Notably, additional work shows that the activity of DNA–PK, a complex between the Ku70/80 heterodimer and DNA-PKcs (see later in the text), is also inhibited by short (14–20 bp) DNA fragments (102).

Two essential processes for the maturation of the immune system are mediated by the programmed and highly regulated induction of clusters of DSBs, and in both processes, the intervening DNA segment is lost, albeit in a highly regulated manner (10,104). In V(D)J recombination, taking place in developing B (and T) lymphocytes, the N-terminal variable region of Ig heavy and light chains that bind the antigen is ensembled from germ line V, D and J gene segments. This is achieved by the lymphocyte-specific RAG endonuclease, comprising recombination activating gene (RAG) 1 and 2 proteins. The reaction is initiated by the introduction of two DSBs adjacent to target V, D and J sites and proceeds with the removal of the intervening DNA segment and the joining of remaining DNA ends by non-homologous end-joining (NHEJ). Subsequently, and on antigen activation, mature B cells also undergo IgH CSR that replaces one set of IgH constant region exons with another, allowing B cells to secrete different effector antibody classes. CSR is initiated by activation-induced cytidine deaminase that generates DSBs indirectly through clusters of base damage (T4-DSB) in downstream portions of IgH. Such DSBs are joined by NHEJ to complete CSR (105). Notably, this generation of functional antigen receptor loci via clustered-DSB intermediates poses great oncogenic risks (106), which are compounded by the ability of antigen receptor locus regulatory elements to activate expression of the translocated oncogene.

**Figure 5.**
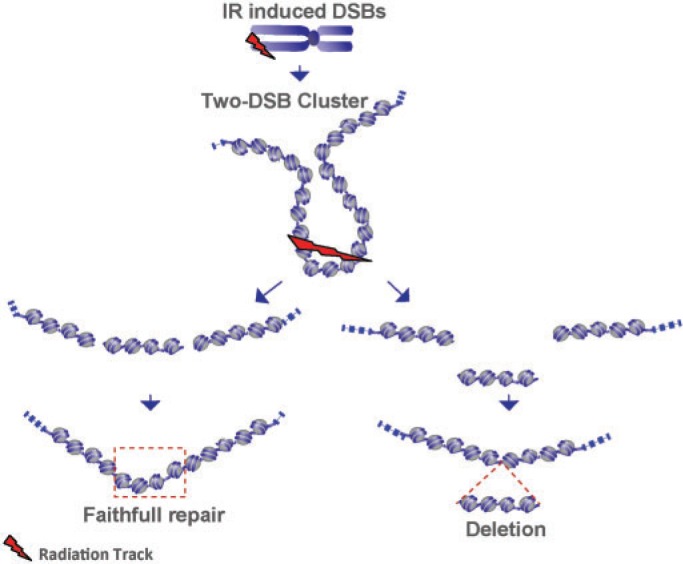
Fragment loss through 2xDSB cluster. An example of clustered DSB: two DSBs in the cluster induced in the linker region between nucleosomes. It can lead to chromatin destabilization through the loss of the DNA segment between the two DSBs. Two possible processing scenarios are illustrated. If the DSB ends stay close, the DNA molecule is restored by simple rejoining. In a second scenario (shown on the right), a small DNA fragment comprising four nucleosomes is lost from the chromatin context causing a deletion and possibly also jeopardizing, or somehow impairing, all forms of processing.

The generation of DSB clusters and their contribution to the adverse effects of IR has also been the subject of extensive mathematical modeling (49). Ostashevsky (107,108) analyzed in this manner the consequences of chromatin fragmentation and ultimately of cell death. The assumption of the developed model is that DSBs generate small and, therefore, unstable DNA fragments (terminal or interstitial) that can be lost from the chromatin context, thus compromising repair of the constituent DSBs (Figures 3 and 5). The probability that such fragments will be lost from their chromatin context is thought to increase with decreasing fragment length. A more specialized induction of DSB clusters within chromatin loops, similar to that considered by Bryant and Johnston, has been used to develop alternative mathematical models by Friedland *et al.* (49,100,101,109), Cucinotta and co-workers (110), as well as by Scholz and co-workers (111–113). The satisfactory fitting achieved under these assumptions of cell survival and DSB repair results suggests that DSB clusters represent a precarious form of DNA damage. Notably, all these models also offer a plausible explanation for the increased biological efficacy of high-LET radiation, as the yields of clustered DSBs are expected to increase, and the length of the associated fragments to decrease with increasing LET (see later in the text). An example of clustered DSBs generating a small (∼10 bp) DNA fragment is shown in Figure 4 for the energy deposition pattern calculated for the α particle.

In aggregate, the aforementioned work provides strong albeit indirect support for DSB clustering as yet another level of DSB complexity, which we here term type 6 DSBs—T6-DSBs (Figure 3F). Notably, this form of DNA damage is only rarely studied experimentally despite its potential implications in the adverse effects of IR. Repair complications from DSB clustering will mainly derive from the instability of the generated DNA fragments, whose loss from the higher-order chromatin context is likely to impair the function of all DSB repair pathways (Figure 5) and to cause thus chromosome aberrations (114). Similar complications in repair may incur during chromothripsis and the consequences of the associated chromosome shattering observed may have the same mechanistic underpinnings as those of IR-induced DSB clusters (90–93).

The probability of fragment loss from DSB clusters is likely to depend on the distance between constituent DSBs but will also be strongly determined by the structure of chromatin and its degree of condensation at the cluster site (see the two examples illustrated in Figure 3F involving only one nucleosome, or a larger chromatin segment). Although the prevailing model for chromatin structure >10-nm nucleosome filament is that of a 30-nm chromatin fiber including 6–7 nucleosomes per 10-nm length of fiber, recent results question the existence of such structure (115–119). The characterization of human chromatin using novel chromosome conformational capture techniques (23,120–124) favors an alternative structural model of human chromosome with the 10-nm fiber folded in a regulated manner as a long-lived fractal globule—a compact polymer state that emerges during polymer condensation as a result of topological constraints, which prevent one region of the chain from passing across another one (Figure 3, lower right model) (36,37). It will be particularly interesting to examine the stability of DNA fragments generated by DSB clusters in this model of chromatin architecture.

One limitation of the approaches taken hitherto to understand the consequences of T6-DSBs is that they are indirect, and only mathematical modeling allows connection to biological consequences (49). Vice-versa, approaches documenting the formation of such DNA fragments are in general devoid of directly linked biological effects. As a result the conclusions drawn are tentative and indicative at best.

The nature of DSB induction precludes mechanistic experiments on T6-DSBs using IR as a model agent, as each of the irradiated cells sustains DSBs in a stochastic manner at different numbers and severity, which are randomly distributed throughout the genome; thus, analysis of effects is possible only by theoretical modeling that is tested by fitting to existing data (49). The earlier discussed uncertainty about the 30-nm chromatin fiber that implicitly or explicitly underpins present modeling approaches further complicates the situation. The field will benefit from molecular biology approaches modeling defined combinations of DSB clusters and testing their effects. For example, cell lines can be developed in which simple DSBs, and DSB-clusters are generated by restriction of I-SceI recognition-sequence-clusters (or the sequences of other site-specific restriction endonucleases) engineered *in vitro* at defined distances (or designed to cut at specific locations in the genome) in a plasmid that is subsequently integrated in multiple copies in the cellular genome. We are presently testing and validating this approach in our laboratory.

## PATHWAYS OF DSB REPAIR AND THEIR INHERENT PROPENSITIES FOR PROCESSING ERRORS

Key components of DDR are evolutionarily conserved repair pathways processing DSBs to preserve the integrity of the genome (125,126). DSB repair pathways are broadly classified as homology dependent and homology independent. Homology-independent pathways function throughout the cell cycle and include the DNA-PK–dependent non-homologous end-joining (D-NHEJ; the terms classical or canonical are also frequently used to describe this repair pathway), as well as an alternative end-joining pathway that under certain circumstances operates as back-up to D-NHEJ, and possibly also to homologous recombination repair (HRR), and is, therefore, termed alt-EJ, or B-NHEJ. Homology-dependent pathways, on the other hand, show strong cell cycle dependence and operate only when a sister chromatid becomes available after semi-conservative DNA replication. In the following sections, we describe the key features of each of these DSB repair pathways, outline their inherent propensities for errors and describe the types and sources of errors they can produce.

### Homologous recombination repair

**Figure 6.**
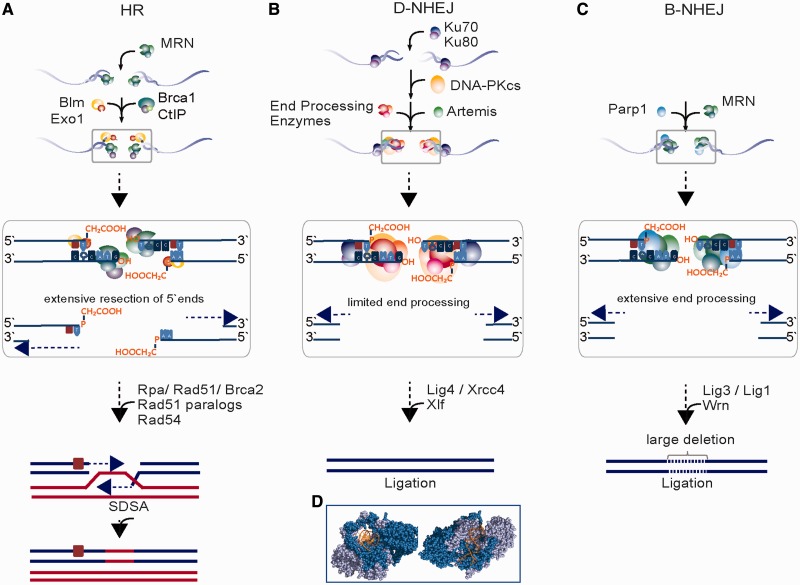
Key steps of DSB repair pathways (HRR, D-NHEJ and B-NHEJ) with examples of end-processing options for T3-DSBs. (**A**) During HRR, extensive processing of the 5′-ends takes place that can remove lesions in the vicinity of DSB ends. Although base damage remains at the 3′-end after HRR, the DSB is repaired and the remaining single base lesion can be removed by BER at a later time. (**B**) For D-NHEJ, limited end processing takes place at both DNA strands—5′ and 3′. As a result, lesions that span up to 10 bp from the DSB ends could be removed as well, although their presence is likely to delay this processing. (**C**) During B-NHEJ, even more extensive end processing takes place, and as for D-NHEJ, lesions adjacent to the DSB may be removed. B-NHEJ often results in large deletions—and translocations. (**D**) Illustration of Ku bound to DNA. This protein–DNA interaction was visualized using the program PyMOL (PyMOL Molecular Graphics System, Version 1.2r3pre, Schrödinger, LLC). The results are from (129). The structure illustrates that each Ku molecule binds roughly two helical turns of DNA.

HRR is an error-free repair process (127,128) that can be divided into three main stages: pre-synaptic, synaptic and post-synaptic (Figure 6A). After sensing of the DSB by MRN (Mre11-Rad50-Nbs1) in the pre-synaptic stage, the DNA is resected at the DSB site to form an extended region of single-stranded DNA (ssDNA) with 3′-overhangs. Several factors have been implicated in this step including MRN, Exo1, Dna2 and CtIP, as well as the BLM helicase (15). The ssDNA generated in this way is promptly coated by RPA for stabilization from secondary structures and preparation for Rad51 nucleoprotein filament formation. For efficient Rad51 filament formation, different classes of mediator proteins like the Rad51 paralogs (Rad51B, Rad51C, Rad51D, Xrcc2 and Xrcc3), as well as Brca2 are used.

During synapsis, the Rad51 nucleoprotein filament searches for homology and performs strand invasion to form a Holliday junction. Rad54 promotes DNA synthesis associated with branch migration by dissociating Rad51 from the heteroduplex DNA. In the post-synaptic steps associated with a specific sub-pathway (130), synthesis-dependent strand annealing (SDSA), the extended Holliday junction is resolved. This enables the annealing of the newly synthesized strand with the resected strand of the second DNA end and restores the broken DNA molecule by subsequent DNA synthesis and ligation (Figure 6A).

SDSA is a common DSB repair mechanism in cells of higher eukaryotes. In a different sub-pathway of HRR (not depicted), invasion of both DSB ends into the non-damaged sister chromatid leads to the formation of a double Holliday junction that migrates along the chromatids by DNA synthesis; its subsequent resolution is associated with crossover or non-crossover events depending on which strands are cut by a resolvase (130,131). The outlined complexity of the events involved in HRR and the requirement for homology search makes this repair pathway inherently slow.

The templated nature (through the sister chromatid) of DSB-repair by HRR not only ensures the structural restoration of the DNA molecule but also enables the preservation of the DNA sequence at the DSB. As a result, HRR is an error-free repair pathway on every count.

The events initiating HRR imply that a wide spectrum of structural DNA-end substrate configurations at the DSB, like variations in the overhang length, DNA-end sequence and DNA-end chemistry (e.g. 3′-phosphoglycolate or 5′-OH present in T2 and T3-DSBs) can be accommodated, although they may slow processing (see earlier in the text). This is because many of the altered or missing bases will be removed during resection, and those present in the 3′-ends that are not resected may be either removed by limited resection or may remain in the DNA for processing after completion of the DSB repair (illustrated in Figure 6A). HRR can thus function as a processing integrator for DSB ends with widely different chemistry. We return to this flexibility later in the text.

### D-NHEJ

D-NHEJ is widely considered as the prevalent DSB repair pathway in higher eukaryotes (125,126). It mediates the fast ligation of broken DNA ends to ensure chromosome integrity (16) (Figure 6B). It is initiated by the binding of the Ku70/Ku80 heterodimer to DSB termini, which in-turn recruits and activates the large protein kinase, DNA-PKcs, to generate a binding scaffold for other NHEJ factors and to mediate their regulation by phosphorylation (132). The process culminates with the ligation of the two DNA ends by the Ligase 4/Xrcc4/Xlf protein complex after displacement of DNA-PKcs from the ends through autophosphorylation. When required, various DNA end-processing functions, including the addition of a 5′-phosphate by Pnk and the removal of 3′-phosphoglycolates by Tdp1, Pnk or Artemis, ensure the generation of ligatable DNA ends (69). Filling of occasionally missing nucleotides is mediated by DNA polymerases λ and µ.

The earlier outlined mechanistic background of D-NHEJ directly points to important strengths but also indicates inherent limitations. D-NHEJ enzymes tolerate a wide spectrum of structural DNA-end substrate configurations, like variations in the overhang length, DNA-end sequence and DNA-end chemistry. It thus can also function as an important integrator funneling for processing ends with widely different chemistry.

The second important feature of this pathway is speed of operation (13). The DSB kinetics shown in Figure 1 actually reflects the function of this repair pathway. Although not formally shown, the key factors of this pathway likely operate in unison and form through sequential interactions a molecular machine at the DSB that ensures fast repair. This unparalleled speed (Figure 1) may be the most defining characteristic of D-NHEJ, as it also maximizes the probability for the joining of the original DNA ends—by reducing the time available for diffusion of DNA-ends away from each other (13). As a result, D-NHEJ suppresses chromosome translocations (13,14,133,134). However, as far as we know at the moment, this pathway has no build-in means (possibly apart from the efficiency of the associated molecular machine) to ensure joining of the original DNA ends- or to suppress joining of incorrect ends. Thus, translocations are in principle possible and do occur through this repair mechanism, albeit infrequently.

Notably, the most salient limitation of the pathway is the absence of build-in mechanisms ensuring the restoration of DNA sequence at the DSB. As a result, changes in nucleotide sequence, or additions and deletions of nucleotides, are likely events (16). Such events become far more likely when end-processing is required to generate ligatable ends, as it is, for example, the case for DSBs of types 2–5. However, here again the high speed of operation ensures that the processing occurring at the ends is more limited than after end joining by the alternative pathway discussed later in the text.

**Figure 7.**
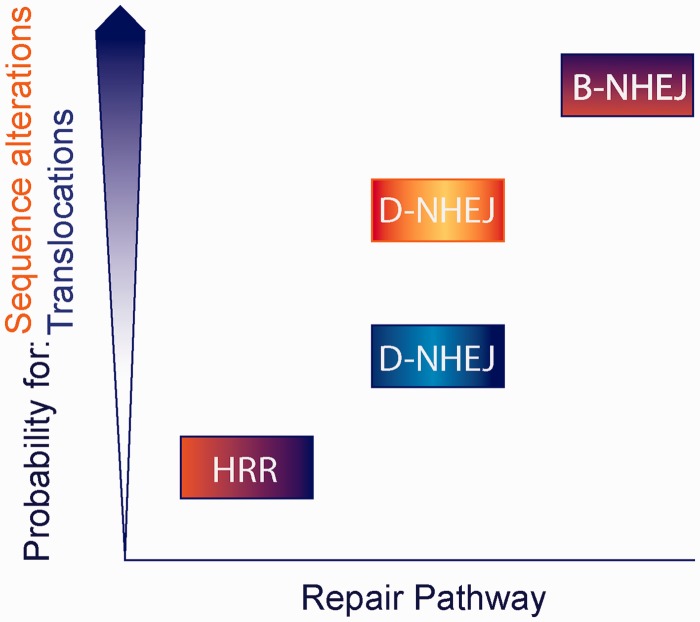
Propensity for errors by HRR, D-NHEJ and B-NHEJ. For each repair pathway, the probability for sequence alterations at the junction is indicated with orange shading, whereas the probability for translocations is indicated with blue shading. The scale is arbitrary and serves only illustration purposes—also when comparing the two sources of errors. HRR has very low probability for both sequence alterations at the junction, as well as for translocations. D-NHEJ has low probability for translocations, but relatively high probability for sequence alterations at the junction. B-NHEJ is, on the other hand, highly error prone on all counts.

These circumstances render D-NHEJ inherently error prone with high probability for sequence alterations at the junction but low probability for translocations (Figure 7). Indeed, analysis of sequence alterations after RE-induced DSBs at the endogenous thymidine kinase gene (TK) revealed deletion sizes from 1 up to 1201 with a median deletion size of 22 bp (135). Finally, and in line with the arguments raised earlier in the text, damaged-end groups such as damaged bases and sugars, do not affect DNA-PK activation, suggesting that end group chemistry does not have an inhibitory effect on DNA-PK kinase activity (102), although there is evidence for processing impairment *in vitro* assays (73,76).

### B-NHEJ

B-NHEJ is an alternative form of DNA end-joining thought to function as back-up to D-NHEJ (13), and possibly also to HRR, hence, the term B-NHEJ (Figure 6C). However, the term alternative end-joining is also frequently used (105,126,136). Although it functions on similar principles, B-NHEJ is slower and less efficient and as a result more error prone than D-NHEJ on two counts (Figure 7). First, deletions and other modifications at the junction are larger than after D-NHEJ. Second, and particularly relevant, the joining probability of unrelated ends is markedly increased. Thus, although the differences in the type of errors generated by D-NHEJ and B-NHEJ are quantitative rather than qualitative (both are unable to restore the junction and can join unrelated DNA ends), B-NHEJ is considered a main source of chromosomal translocations (Figure 7) (13,14,133,134).

Although B-NHEJ can be conveniently studied when D-NHEJ is genetically or chemically compromised, B-NHEJ is thought to get engaged in all cases where D-NHEJ, and possibly HRR, somehow fails. Such failures may include all instances where the assembly of the aforementioned D-NHEJ machine at the DSB is unsuccessful. It seems that B-NHEJ can function in the presence of certain D-NHEJ components (e.g. Lig4, DNA-PKcs etc. in Ku-deficient cells), but it is not clear what role these remaining D-NHEJ components play in the rejoining process. On the other hand, there is evidence that other D-NHEJ components, e.g. Ku, suppress B-NHEJ by preventing one of its putative components, Parp-1, to bind to DNA ends (137).

Although the enzymology and mechanistic details of B-NHEJ are incompletely understood, there is evidence that this pathway can use DNA ligases I and III (Lig1 and Lig3) in the final step (Figure 6C) (138–141). The involvement of Lig3 also explains the involvement of the Parp-1/Lig3/Xrcc1 module that is known to be involved in the repair of SSBs (125,141,142). However, recent work questions the requirement for Xrcc1 in DSB repair by this pathway (138,139,143).

Probably as a result of the slow kinetics but certainly also by virtue of its functional characteristics, B-NHEJ allows more DNA end processing than D-NHEJ. Not surprisingly, therefore, a number of end-processing activities involved in HRR, such as Mre11 (144) and CtIP, as well as Wrn and Bcr/Abl have been implicated in its function (145). This is in line with the possibility that B-NHEJ also backs up failed HRR and explains the frequent presence of microhomologies at B-NHEJ-mediated junctions (136). However, in several experimental systems, microhomology is not a requirement for efficient B-NHEJ. Finally, B-NHEJ may benefit from the linker histone H1 serving as an alignment factor (146).

Like D-NHEJ, B-NHEJ is also active throughout the cell cycle (147–150). However, unlike D-NHEJ, it shows strong cell cycle-dependent fluctuations with increased activity in G_2_, reduced in G_1_ and markedly ablated in resting cells (147,148,151,152). Like the other DSB repair pathways, B-NHEJ can accommodate a wide spectrum of DNA-end chemistries (Figure 6C).

### Analysis of processing complications for T1-T6-DSBs

The earlier outline indicates that IR-induced DSBs can be present in different ‘flavors’, which in the case of IR strongly depends on the energy deposition events underlying their induction and, thus, the type of radiation used to generate them. DSB complexity, as outlined earlier in the text, is likely to confound DSB processing and may increase the risk of generating processing failures as outlined in Figure 2.

T1-DSBs can be shunted to all known DSB repair pathways, and there are no complications associated with the repair by anyone of them beyond the destabilization of the molecule generated by the DSB and which is a common characteristic of all DSBs.

T2-DSBs have non-ligatable ends and will require end processing before the final ligation. This end processing should be straightforward when repair is started by HRR because of the extensive end processing integrated in this repair pathway (Figure 6A). End processing is also an integral part of D-NHEJ and should also be efficient in B-NHEJ as indicated by the high speed it processes a relatively large proportion of DSBs in D-NHEJ-deficient cells (Figure 6B and C).

Investigation of the processing of T3 and T4 DSBs is a highly active area focusing on the consequences of damage clustering on the functions of enzymes involved in base damage and strand break repair (30,70,153).

But, is the increased level of complexity of T3 and T4 DSBs expected to compromise DSB processing by the known repair pathways? DSB processing by D-NHEJ starts with the binding of Ku to the generated DNA ends, an interaction that occurs extremely fast (10^−^^9 ^M) and involves >15 bp of sequence on each side of the break (Figure 6D) (129). Ku binding on a T3-DSB will suppress the recruitment of base damage repair factors within a CDS, as it will cover over three helical turns of the DNA—or a 3-fold longer DNA segment than the 10 bp typical extension for a CDS. On the other hand, Ku binding to the DNA ends is not impaired by short single-stranded regions and may not be inhibited by the presence of base damage in the vicinity of the DNA ends ensuring thus normal efficiency for D-NHEJ (132). If the ensuing normal end-processing during D-NHEJ removes the damaged bases of the T3-DSBs, site restoration similar to a T2-DSB will occur—possibly with a slight delay. Alternatively, base damage may be retained and may be removed after the rejoining of the DSB ends. An example of such form of processing is indicated in the insert window of Figure 6B.

Similar arguments can be developed for the processing of T3-DSBs by HRR. Here, resection of the 5′-end for up to 2 kb will remove associated base damage—possibly with only a slight delay. Even base damage present within 10 bp on the 3′-end that is not resected may be removed without grossly impairing subsequent processing steps (see example integrated in Figure 6A). Finally, similar arguments can be developed for the processing of T3-DSBs by B-NHEJ. T4 DSBs are similar to the individual DSBs generated during CSR, and under normal circumstances lymphocytes do not seem to have problems dealing with them. This may also be true to similarly induced DSBs in irradiated cells. There is uncertainty as to how cells will respond to T5-DSBs, but the available evidence suggests that they are detected and processed like any of the previous forms of DSBs (88).

Thus, there are no pathway-specific, urgent reasons rationalizing why T3-T5 DSBs should be much more difficult to repair using HRR, D-NHEJ, of B-NHEJ than T1- or T2-DSBs. This may actually be a reason why the validity of conclusions reached using T1-DSBs as a model has not been so far questioned using more complex forms of DSBs. The question can, therefore, be raised as to whether the most severe form of damage complexity defined here, T6-DSBs, is likely to cause processing problems and thus the adverse effects of IR and their increase with increasing LET. Future work should, therefore, focus on characterizing the consequences of defined T6-DSBs using appropriate systems that allow a conclusive analysis of their biological consequences and test the hypothesis of their increased biological severity.

### General considerations for DSB repair pathway choice

The preceding description of the characteristics of the known pathways engaged in the processing of DSBs indicates marked differences in their inherent ability to faithfully repair the DSB and thus to maintain genomic integrity. In a hierarchical categorization of the pathways, B-NHEJ will have the highest propensity for errors and HRR the lowest (Figure 7). Actually, among the available repair pathways, only HRR is designed to restore every aspect of a DNA molecule that has sustained a DSB. Yet, HRR can only function when a sister chromatid is present, and even then it is bound to be slow. D-NHEJ, on the other hand, functions throughout the cell cycle, including S- and G_2_-phase and has the potential to quickly remove DSBs from the genome, and thus to structurally stabilize it. However, as pointed out earlier in the text, this speedy stabilization has its price, as D-NHEJ readily accepts sequence information losses at the junction. Also the joining of unrelated ends is possible and can lead, although infrequently, to chromosome translocations (Figure 7).

B-NHEJ surfaces as the most precarious of all DSB repair pathways, as it combines increased level of information loss at the junction with much higher probability for chromosome exchange formation. As in some experimental settings, NHEJ pathways seem to be preferred over HRR in cells of higher eukaryotes (see earlier in the text), one can speculate that cells have developed tolerance for DNA sequence modifications at the junction. The same tolerance mechanisms likely allow chromosome exchanges, thus causing cell death, and in multicellular organisms cancer (14,133,134).

Thus, we become confronted with the conundrum that documented errors in DSB processing, with severe adverse effects, are inherent to the repair pathways used by the cells. This begs the question why cells chose an error-prone repair pathway when an error-free repair pathway, HRR in this case, is available and functional—at least in G_2_ (154). It also directly points to issues that need to be addressed when analyzing the network of processes and the decisions that underlie repair pathway choice (15,130,155–157).

This question is particularly relevant because choice among HRR, D-NHEJ or B-NHEJ cannot be considered as one among equivalent options, all of which will lead to the same outcome, i.e. the restoration of the DNA molecule. Rather, different outcomes are certain depending on the choice made and the risk of errors will also be widely different—possibly by orders of magnitude. It would appear logical, at least for cells in G_2_ and S-phase to always first attempt repair by HRR and to opt for alternatives only when this pathway fails to engage. Even then D-NHEJ should be considered the first choice with B-NHEJ remaining as last resort—like all back-ups. Within this rationale, the acceptance of error-prone repair pathways will be a compromise taken only after error-free repair pathways failed. Such sequence of priorities would best satisfy the ultimate goal of preserving genomic integrity and accepting errors only to avert the most severe consequences associated with complete lack of repair.

However, this apparently logical scenario does not seem to form the basis of the detectable cellular response, as the extremely high affinity of Ku for DNA ends is likely to initiate D-NHEJ in the vast majority of DSBs. It also leaves unanswered the question as to whether G_1_ cells or S-phase cells sustaining DSBs in unreplicated segments of their genome, completely lack means to faithfully restore their genome. These apparent inconsistencies point perhaps to gaps in our knowledge regarding the parameters determining DSB repair pathway choice and the logic underlying this choice.

## CONCLUSIONS

DSBs are removed with extremely fast kinetics from the genome. Therefore, repair difficulty cannot be invoked to explain their devastating consequences for the cells. Rather, DSB repair is associated with a high probability for errors, and actually the probability for errors is for DSBs much higher than for any other DNA lesion. Indeed, the adverse biological effects of DSBs derive in their majority from errors in the processing of only few of them. Three pathways process DSBs using different concepts and being associated with different probabilities for errors. This inherent inequality in features and error-risks generates important questions regarding the logic behind repair pathway choice. Are repair pathways engaging DSBs on a first-come-first-serve basis, i.e. as winners of a competition? If yes why? Is this the best way to decide? If not what logic underlies the selection? HRR is the only, in principle, error-free repair pathway. NHEJ pathways are likely to cause sequence alterations at the DSB junction and translocations; both risks are highest for B-NHEJ. The spectrum of DSBs with their increasing complexity further complicates the substrate fed into the repair pathways and must be considered as a key determinant of the risk for errors. Analysis of the spectrum of possible DSB types leads to T6-DSB, representing DSB clusters (local chromothripsis), as the potentially more dangerous of all. The development of defined biological systems allowing examination of severity of different types of DSBs is highly desirable.

## FUNDING

‘Bundesministerium für Bildung und Forschung’ [BMBF: 02NUK005C and 03NUK001B]; ‘Bundesministerium für Wirtschaft und Technologie’ [BMWi: ESA-AO-08-IBER, 50WB1229]. Funding for open access charge: Bundesministerium für Bildung und Forschung Germany.

*Conflict of interest statement*. None declared.
